# The burden of epilepsy in the People’s Republic of China from 1990 to 2019: epidemiological trends and comparison with the global burden of epilepsy

**DOI:** 10.3389/fneur.2023.1303531

**Published:** 2023-12-11

**Authors:** Yun Shu, Zhifeng Wu, Xiaolin Yang, Min Song, Yangyang Ye, Chunqing Zhang, Qing Yuan, Li Wang

**Affiliations:** ^1^Medical College of Acu-Moxi and Rehabilitation, Guangzhou University of Chinese Medicine, Guangzhou, China; ^2^Department of Pediatrics, Second Affiliated Hospital, Army Medical University, Chongqing, China; ^3^National Comprehensive Epilepsy Center, Department of Neurosurgery, Second Affiliated Hospital, Army Medical University, Chongqing, China; ^4^Department of Neurology, Second Affiliated Hospital, Army Medical University, Chongqing, China

**Keywords:** epilepsy, global burden of disease, jointpoint regression, age-period-cohort model, social demographic index

## Abstract

**Background:**

Epilepsy is associated with a significant global burden of disease, affecting over 50 million people worldwide. The specific aim of this study is to compare the burden of epilepsy in the People’s Republic of China (PRC) with the global burden, and to analyze the epidemiological trends of epilepsy, the relationship between the burden of epilepsy and social demographic index (SDI), and the relative contributions of epidemiological factors.

**Methods:**

This is a retrospective population-based study, data were obtained from the Global Burden of Disease (GBD) study in 2019. We employed Joinpoint software and the age-period-cohort (APC) model to analyze epilepsy’s epidemiological trends. Health inequality analysis was conducted to investigate the impact of SDI on epilepsy burden. Decomposition analysis was performed to examine the relative contributions of age, population, and epidemiological changes to epilepsy.

**Results:**

Between 1990 and 2019, the incidence rate in the PRC increased by 45%, significantly surpassing the global incidence of epilepsy. However, Disability-Adjusted Life Years (DALY) decreased notably, and the proportion of Years of Life Lost (YLL) decreased from 62.73 to 39.03%. Concerning incidence, the period Rate Ratio (RR) in the PRC initially increased and then decreased, while the cohort RR in the PRC and globally exhibited a consistent upward trend. In terms of mortality, period RR and cohort RR in the PRC displayed a gradual decrease, with mortality starting higher but eventually falling below the global mortality. The net drifts of incidence were greater than 0, whereas the net drifts of mortality were less than 0, both were lower in the PRC than at the global level. Decomposition analysis indicated that the changes of incidence and mortality in the PRC were mainly attributed to epidemiological changes. Additionally, global disparities in epilepsy decreased, with the burden concentrating in low SDI countries.

**Conclusion:**

The incidence of epilepsy in the PRC rose during the 30-year study period, while epilepsy mortality decreased. The improved survival rate in the PRC is predominantly attributable to epidemiological changes. The burden of epilepsy in the PRC predominantly affects males, children, and the elderly, Chinese government should focus on specific populations.

## 1 Introduction

Epilepsy is a chronic cerebral disease characterized by persistent epileptic tendencies, leading to recurrent seizures and transient neurological dysfunction attributed to abnormal neuronal activity (1). Epilepsy can be categorized based on etiology into idiopathic epilepsy, genetic epilepsy, infectious epilepsy, metabolic epilepsy, structural epilepsy, and immune epilepsy (2). The Global Burden of Disease (GBD) study in 2019 reported that epilepsy in the People’s Republic of China (PRC) accounted for 10% of global disability-adjusted life years (DALY) and 94% in East Asia (3). Worldwide, over 50 million people suffer from epilepsy, with more than 2 million new cases diagnosed annually, and 80% of patients residing in low and middle-income countries (4, 5). Epidemiological surveys have revealed that the PRC has nearly 10 million epilepsy patients, with 400,000 to 600,000 new cases reported each year (6, 7). Epilepsy remains a significant health concern in the PRC, particularly in its western provinces. The incidence and prevalence of epilepsy increased from 1990 to 2019, with a steady rise in the burden among the elderly (8). People with epilepsy often experience negative psychological and social impacts, including anxiety, depression, low self-esteem, learning disabilities, unemployment, and lower marriage rates. Epilepsy is also associated with a mortality rate 3 to 10 times higher than that in the general population and contributes to various causes of death (9, 10), including direct causes (such as accidental death and persistent status epilepticus) and epilepsy-related causes (such as suicide and complications of epilepsy treatment) (11, 12). Consequently, epilepsy represents a substantial burden on both families and society, constituting a significant public health challenge.

The GBD study, compiled and published by the University of Washington in the United States, serves as an indicator of the health and economic impact of diseases, injuries, and premature deaths within populations and countries (13, 14). Previous reports on the burden of epilepsy in the PRC have often used outdated GBD data or focused on other countries or regions, lacking a comprehensive comparison between the PRC and the global situation as of GBD 2019. Furthermore, previous studies have not undertaken health inequality analysis or decomposition analysis of epidemiological factors (15). In this study, we utilized the latest GBD 2019 data to examine and compare the burden of epilepsy in the PRC and globally, assessing age groups, gender disparities, and temporal trends over the past three decades. We employed Joinpoint regression and the age-period-cohort (APC) model to ascertain incidence and mortality trends and to assess the impact of socioeconomic factors on epilepsy burden. Health inequality analysis was employed to investigate the influence of SDI on epilepsy burden, while decomposition analysis was conducted to determine the relative contributions of age, population, and epidemiological changes to epilepsy. Our findings aim to inform evidence-based national policies, resource allocation, and health system planning to alleviate the epilepsy burden.

## 2 Materials and methods

### 2.1 Data sources

We utilized data from GBD 2019, which provided the complete burden metrics only for idiopathic epilepsy. Idiopathic epilepsy was defined by the GBD study according to the ILAE proposal in 1985 (16). Thus, the main analysis specifically focusing on idiopathic epilepsy where identifiable structural or metabolic causes were absent, and only included those of genetic or unknown causes (6, 17). Accessing the GBD visualization database, maintained and regularly updated by the Institute for Health Metrics and Evaluation (IHME) at the University of Washington, we employed the GBD Result Tool for database searches. GBD 2019 comprises comprehensive data concerning the global burden of disease for 369 conditions and 87 risk factors across 204 countries and regions spanning the period from 1990 to 2019. This data is categorized by region, year, sex, and age (18, 19). The GBD data were sourced from the official website of the Global Health Data Exchange Center,^1^ recognized as the world’s largest health data repository (20). Data pertaining to the PRC of various diseases in GBD 2019 were derived from diverse sources, including demographic surveys, national censuses, disease surveillance point systems, and death cause registration report information systems (21). We conducted a secondary analysis of GBD 2019 by implementing a systematic review utilizing spatiotemporal Gaussian process regression and DisMod-MR V.2.1 (a Bayesian meta-regression tool) methods (18, 22). All calculations in GBD 2019 were repeated 1,000 times, and the upper and lower bounds of the confidence interval (CI) were defined by the 25th and 975th values among the ranked estimates (23, 24). A significant difference was observed if 975 or more of the 1,000 variance values fell on one side of zero. To account for uncertainties related to measurement error, bias, and modeling, all estimated values were presented with a 95% confidence interval (95% CI), ensuring a comprehensive evaluation of epilepsy burden at specific time points. The age-standardized rate (ASR), based on the world standard population developed by GBD 2019, was calculated as a weighted average of age-specific rates, allowing for the elimination of age-related effects and facilitating comparisons across sex, age, and year.

Based on epidemiological and demographic indicators, we extracted data on the number and rate of prevalence, incidence, death, DALY, Years of Life Lost (YLL), and Years Lived with Disability (YLD) from GBD 2019, with a primary focus on the incidence and mortality of epilepsy. All indicators were reported as the value, the rate per 100,000 individuals, and age-standardized rates per 100,000 individuals. Incidence or mortality denote the ratio of new cases or deaths to the total population in a given year. DALY represents an integrated metric for estimating disease burden, including all healthy life years lost from the onset of the disease until death. It offers a comprehensive measure of both the quantity and quality of life over time (11, 25). DALY is the sum of YLD and YLL. YLD accounts for individual disease sequelae’s prevalence multiplied by a disability weight (ranging from 0 to 1, where 0 represents full health and 1 equals death), reflecting the number of years lived with disability due to the disease. YLL represents the number of deaths multiplied by the standard life expectancy at the time of death, as per the GBD 2019 standard life table. YLL quantifies the years of life lost due to premature mortality.

### 2.2 Joinpoint regression analysis and age-period-cohort model

Joinpoint regression analysis was employed to analyze long-term disease trends, with data adjustments incorporating three interacting factors: age, period, and cohort effects, within the age-period-cohort (APC) model. The model accounted for all confounding factors and allowed for the independent analysis of age, period, and birth cohort effects. Given the complete collinearity of the model (cohort = period – age), we organized data into five consecutive years for age, period, or cohort groups (26). We applied the Intrinsic Estimator (IE) in the analysis process (27). The APC model’s formula was expressed as follows: Y = log (M) = μ + αX1 + βX2 + γX3 + ε, with X1, X2, and X3 representing age, period, and birth cohort groups, respectively; M representing disease incidence or mortality; α, β, and γ denoting the estimated age, period, and birth cohort effect values, respectively; μ representing the intercept; and ε representing random error following a normal distribution (28–30).

We utilized the Joinpoint Regression Program 4.9.1.0 software, developed by the National Cancer Institute of the United States,^2^ to analyze incidence and mortality trends from 1990 to 2019. The analysis employed the Grid Search Method (GSM) and Monte Carlo Permutation Test to optimize the model, with Bonferroni correction to maintain the overall asymptotic significance level. The resulting output included the number and location of inflection points and their corresponding *p*-values (27, 31–33). This model provided a valuable parametric structure while complementing standard nonparametric description methods. It allowed for localized variations while fitting the overall disease trend and employed logarithmic linear regression to calculate the average annual percentage change (AAPC) and its 95% confidence interval (CI). The annual percentage change (APC) of the slope within a specific interval followed this relationship: APC_i = (exp (beta_i) – 1) * 100. AAPC, weighted by each segment’s duration, represented the weighted sum of APCs for all segments, as indicated by the formula: AAPC = (exp(sum(beta_i * W_i)/sum(W)) – 1) * 100 (27, 34–36). APC > 0 indicated an increasing trend, APC < 0 indicated a decreasing trend, APC = 0 indicated no change, and APC = AAPC indicated a monotonically increasing or decreasing trend. Additionally, the estimated annual percentage change (EAPC) was calculated for age-standardized incidence rates, stratified by sex, region, and cause. The formula for EAPC was expressed as γ = α + βχ + ε, where γ = ln (ASR), χ represented the calendar year, ε indicated the error term, and β characterized the trend as either negative or positive in the age-standardized rates (37, 38). EAPC values greater than 0, along with their 95% CIs, indicated an upward trend, while values less than 0 indicated a downward trend.

The APC model was utilized to assess the effects of age, period, and birth cohort. The age effect was represented as a longitudinal age curve, adjusted for period deviations, and fitted to the selected cohort (27). The period effect was quantified as the period rate ratio (period RR), indicating the risk ratio of a specific period relative to a reference period, adjusted for age and cohort effects (34). The cohort effect was expressed as the cohort rate ratio (cohort RR), signifying the risk ratio of a particular birth cohort in comparison to a reference cohort, adjusted for age and period effects (4). RR values greater than 1 indicated a higher relative risk for the period or cohort compared to the reference period or cohort, while RR values less than 1 indicated a lower relative risk. Local drift represented the logarithmic linear cycle and birth cohort for the respective age group, reflecting the annual percentage change specific to that age group (39). Net drift indicated the overall logarithmic linear trend and cohort effect, representing the total annual percentage change, weighted by the annual percentage change of each segment (39).

### 2.3 Health inequality analysis and decomposition analysis

The social demographic index (SDI) serves as a comprehensive measure of a country’s socioeconomic development and is a relevant health predictor. It combines indicators such as *per capita* income allocation, average educational attainment among individuals aged 15 and older, and the total fertility rate for individuals under 25 (40). SDI values range from 0 to 1, with higher values signifying greater socioeconomic development. In GBD 2019, SDI was categorized into five levels: high SDI (> 0.805), high-middle SDI (0.690 to 0.805), middle SDI (0.608 to 0.690), low-middle SDI (0.455 to 0.608), and low SDI (< 0.455).

To assess health inequality related to socioeconomic development, we employed the Lorenz curve and computed two common indices: the slope index of inequality (SII) and the relative concentration index (RCI). These indices, respectively, represent absolute and relative health inequality. In our study, we calculated them using DALY rates and the corresponding SDI. SII quantifies the absolute difference between predicted values for the highest and lowest SDI groups, while RCI assesses the degree to which disease burden is concentrated in countries with high or low levels of financial development (41). An RCI of 0 suggests no health inequality, while positive or negative values indicate that disease burden is concentrated in countries with high or low levels of financial development (25, 41, 42).

Considering China’s SDI classification in GBD 2019 (SDI = 0.686) as a middle SDI country, we conducted a decomposition analysis of incidence and mortality (new cases and deaths due to epilepsy) in the PRC, middle SDI countries, and globally. Decomposition analysis is a method to determine how differences in factors contribute to differences in overall values. It helps uncover substantial heterogeneity in demographic and epidemiological trends (38). To quantify the relative contributions of changes in age structure, population size, and epidemiology to the overall epilepsy burden from 1990 to 2019, we utilized Das Gupta’s decomposition method (41). Age structure change primarily reflects population aging, population size change relates to variations in population numbers, and epidemiological change pertains to age and population-standardized incidence and mortality rates (38).

### 2.4 Data analysis

For data analysis, we employed R software (version 4.2.2), Joinpoint (version 4.9.1.0), GraphPad Prism (version 9.4.0), the GBD Result Tool,^3^ the APC model network tool,^4^ and the HEAT plus toolkit for health inequality analysis.^5^ After data cleaning and organization, we used R software and GraphPad Prism to load and install necessary packages such as ggplot2 and maps for data analysis and visualization. A significance level of *p* < 0.05 was considered statistically significant.

## 3 Results

### 3.1 The burden of epilepsy in the PRC and globally from 1990 to 2019

The age-standardized incidence rate of epilepsy in the PRC per 100,000 individuals increased from 17 (95%CI: 11.08 to 23.82) in 1990 to 24.65 (95%CI: 16.74 to 33.56) in 2019, marking a 45% increase. In contrast, the global age-standardized incidence rate increased by only 16.86%. In 2019, the age-standardized prevalence rate per 100,000 individuals in the PRC reached 219.69 (95%CI, 155.39 to 288.28), representing a 35.72% increase compared to 1990. The global age-standardized prevalence rate increased by only 12.98%, indicating that epilepsy incidence in the PRC exceeded the global incidence. Additionally, the decrease in deaths, DALY, and YLL values or rates in the PRC was greater than the global level. Globally, the number of deaths increased by 13.95%, while in the PRC, it decreased by 38.85%. The mortality rate decreased by a factor of 0.2 globally but decreased by a factor of 0.5 in the PRC. In 1990, 62.73% of DALY in the PRC consisted of YLL, indicating that the epilepsy burden in the PRC was primarily due to years of life lost prematurely. In 2019, only 39.03% of DALY consisted of YLL, signifying that the reduction in epilepsy DALY was mainly driven by changes in YLL (Table 1).

**Table 1:** Indicators and percentage change of epilepsy burden in China and globally from 1990 to 2019.

Location	Year	Incidence (95%CI)		Prevalence (95%CI)		Death (95%CI)		DALY (95%CI)		YLD (95%CI)		YLL (95%CI)	
		Cases	Rate per 100,000	Cases	Rate per 100,000	Cases	Rate per 100,000	Cases	Rate per 100,000	Cases	Rate per 100,000	Cases	Rate per 100,000
China	1990	201,012 (129,398,285,347)	17 (11.08,23.82)	1,903,214 (1,326,037,2,587,257)	161.87 (112.81,216.65)	19,183 (16,100,21,994)	1.64 (1.40,1.89)	1,864,626 (1,492,966,2,289,801)	153.44 (122.88,187.17)	694,974 (404,055,1,061,580)	58.56 (34.38,88.75)	1,169,651 (962,380,1,336,840)	94.88 (78.05,108.60)
	2019	305,741 (208,839,409,608)	24.65 (16.74,33.56)	3,078,302 (2,204,838,3,997,316)	219.69 (155.39,288.28)	11,730 (9,982,14,009)	0.77 (0.66,0.91)	1,367,511 (979,924,1,837,608)	99.77 (71.33,133.52)	833,736 (473,712,1,278,491)	60.46 (33.99,93.29)	533,775 (453,358,633,260)	39.31 (33.39,46.87)
	Percentage change (%)	52.10	45	61.74	35.72	−38.85	−53.05	−26.66	−34.98	19.97	3.24	−54.36	−58.57
Global	1990	1,859,517 (1,281,783,2,526,087)	33.22 (23.40,44.69)	15,324,096 (11,446,732,19,630,584)	288.79 (218.16,364.76)	100,054 (81,176, 112,226)	1.94 (1.61,2.15)	11,285,623 (8,614,048,14,136,603)	204.32 (157.63,254.14)	5,388,600 (3,355,711,7,974,011)	99.71 (63.10,146.13)	5,897,022 (4,542,809,6,774,039)	104.61 (81.55,119.21)
	2019	2,898,222 (2,098,718,3,823,376)	38.82 (27.99,51.28)	25,111,110 (19,033,571,31,433,013)	326.27 (247.83,408.33)	114,011 (100,178,129,928)	1.46 (1.28,1.67)	13,077,624 (9,986,730,16,734,086)	170.63 (130.42,218.26)	7,740,804 (4,810,323,11,216,664)	101.13 (63.08,146.83)	5,336,821 (4,722,718,6,170,046)	69.5 (61.69,80.63)
	Percentage change (%)	55.86	16.86	63.87	12.98	13.95	−24.74	15.88	−16.49	43.65	1.42	−9.50	−33.56

DALY, Disability–Adjusted life years; YLD, Years lived with disability; YLL, Years of life lost.

**Figure 1:**
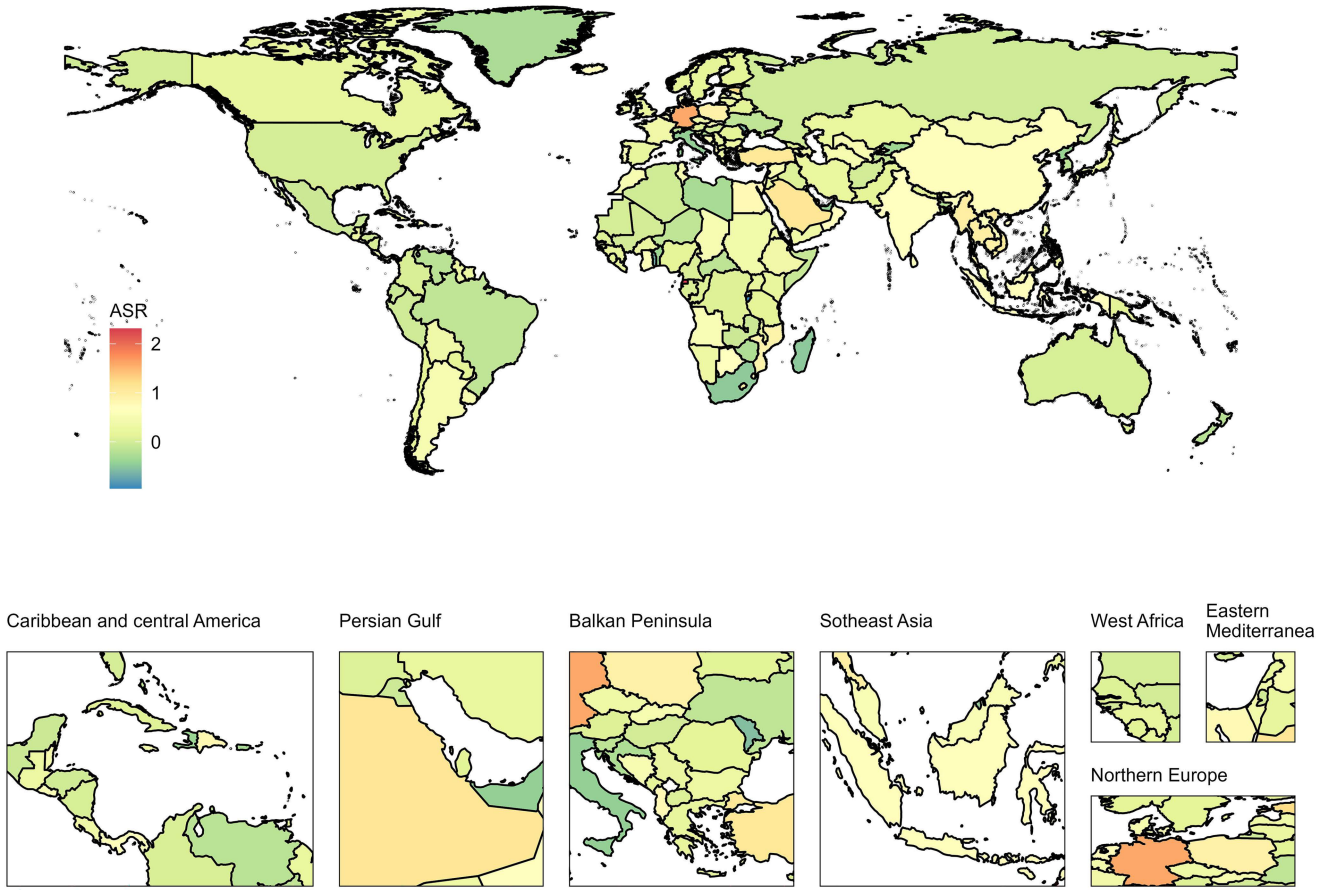
EAPC world map of the age-standardized incidence rate for 204 countries from 1990 to 2019. The color bar on the left refers to the EAPC value from low to high. EAPC, Estimated annual percentage change.

Over the past 30 years, the age-standardized incidence and prevalence of epilepsy in the PRC have increased, while mortality, DALY, and YLL rates have significantly decreased. YLD did not exhibit significant changes, and the burden of epilepsy was higher in males compared to females (Supplementary Figure S1). Both the PRC and the global incidence and prevalence increased by approximately 0.5 to 0.6 times, with the number of incidences far exceeding the number of deaths. The EAPC world map of 204 countries indicated varying degrees of increase in the age-standardized incidence rate of epilepsy across different regions. From 1990 to 2019, China’s EAPC was 0.7% (95%CI: 0.41 to 1%), demonstrating a steadily rising trend (Figure 1). Detailed EAPC values and 95% CIs for all countries are provided in Supplementary Table S1.

### 3.2 Analysis of the incidence trend of epilepsy in the PRC and globally from 1990 to 2019

**Figure 2:**
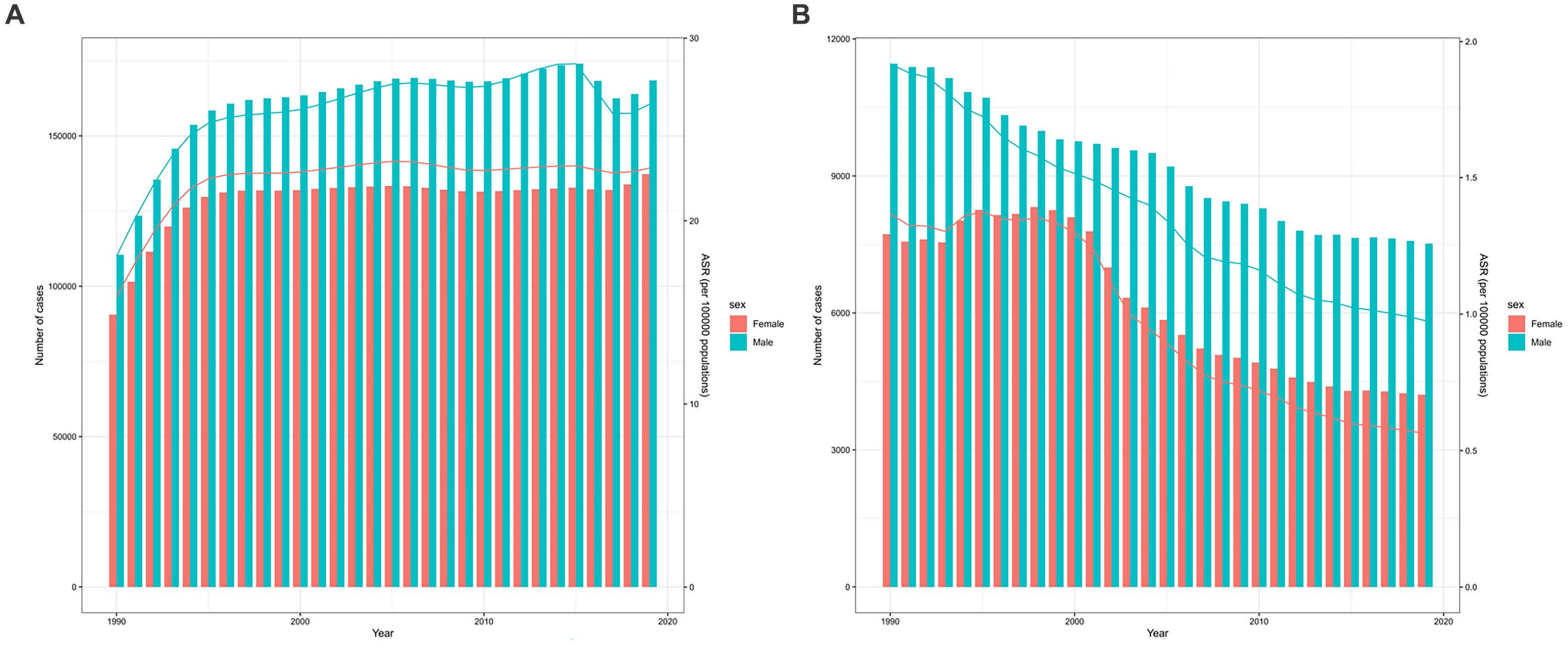
The trends in the number and ASR of the incidence **(A)** and mortality **(B)** in Chinese males and females from 1990 to 2019. The bar graph represents the trend of number, and the line graph represents the trend of ASR. ASR, Age-standardized rate.

The incidence of epilepsy in the PRC exhibits an age-related pattern, with a significant increase in all age groups in 2019 compared to 1990. The peak incidence is concentrated in the 0–9 years age group, and it steadily rises in the elderly, indicating that epilepsy incidence does not necessarily increase with age (Supplementary Figure S2A). In 2019, the age-standardized incidence rate per 100,000 individuals in the PRC stood at 24.65 (95%CI: 16.74 to 33.56), placing it at a middle level when compared to other countries (Supplementary Figure S3A). Both the number of incidences and the age-standardized incidence rate for both sexes in the PRC increased steadily from 1990 to 2019, with the most substantial growth observed during 1990–1994. Incidence and age-standardized rates were higher in males than in females, but the overall trend was quite similar for both sexes. There was a slight decline in incidence and age-standardized rates in males during 2015–2017, followed by another increase. In contrast, females exhibited a relatively stable trend in recent years (Figure 2A).

**Figure 3:**
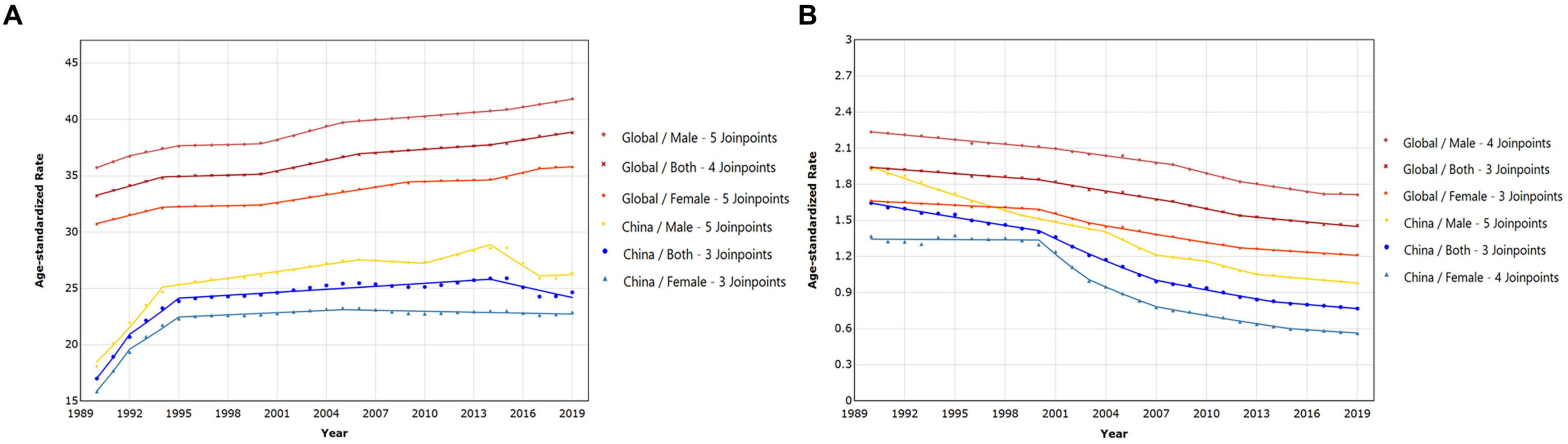
The trends in the time of age-standardized incidence **(A)** and mortality **(B)** rate in different sexes for epilepsy from 1990 to 2019, comparison between China and the world.

Joinpoint regression analysis results indicated that the age-standardized incidence rate in both China and globally increased from 1990 to 2019. The AAPC for China showed an increase of 1.218% (95%CI: 0.787 to 1.651%), while the global AAPC was 0.538% (95%CI: 0.503 to 0.573%), signifying that the increase in the PRC was substantially higher than the global trend. Globally, the incidence rate exhibited a consistent upward trajectory, whereas the PRC reached a peak around 1990–1994 and then remained relatively stable. However, in the last 5 years, there has been a slight decrease in incidence rates for both sexes in the PRC (Figure 3A). Specific APC values for each segment can be found in Supplementary Table S2. The AAPC for Chinese females [1.243% (95%CI: 1.049 to 1.437%)] was slightly higher than that for males [1.215% (95%CI: 0.908 to 1.522%)], while the global average annual growth rate for males was slightly higher than that for females (Table 2).

**Table 2:** The AAPC for the age-standardized incidence and mortality rate in different sexes from 1990 to 2019.

Location	Indicator	Both sexes (95%CI)	Female (95%CI)	Male (95%CI)
China	Incidence	1.218 (0.787,1.651)^*^	1.243 (1.049,1.437) ^*^	1.215 (0.908,1.522) ^*^
	Death	−2.592 (−2.772,-2.412) ^*^	−2.949 (−3.354,-2.544) ^*^	−2.336 (−2.702,-1.969) ^*^
Global	Incidence	0.538 (0.503,0.573) ^*^	0.525 (0.461,0.588) ^*^	0.544 (0.517,0.571) ^*^
	Death	−1.004 (−1.097,-0.910) ^*^	−1.089 (−1.209,-0.968) ^*^	−0.909 (−1.032,-0.787) ^*^

^*^Statistically significant (*p* < 0.05). AAPC, Average annual percentage change.

**Figure 4:**
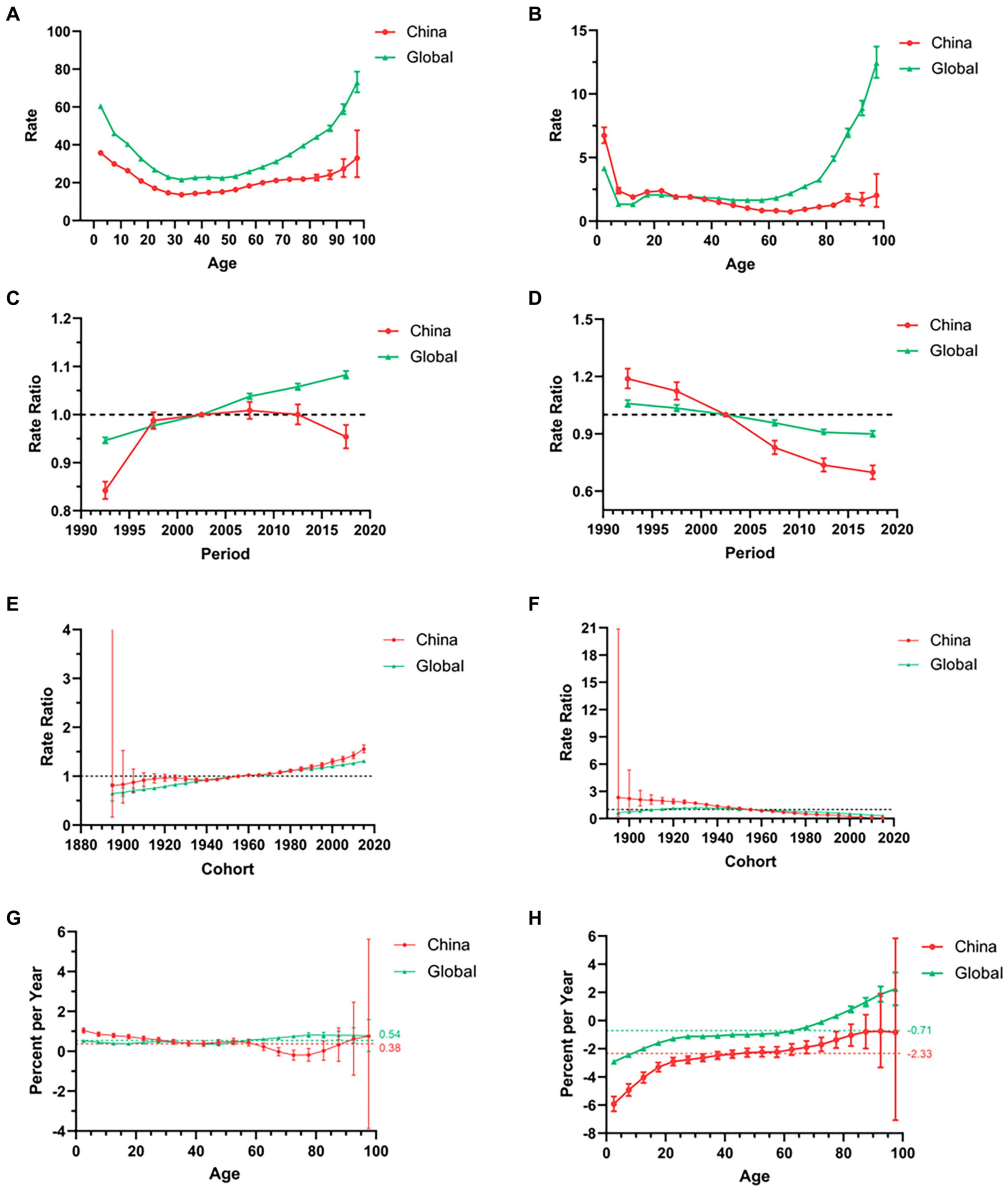
The trends in the incidence **(A,C,E,G)** and mortality **(B,D,F,H)** associated with long age, period RR, cohort RR, local drift, and net drift of epilepsy from 1990 to 2019, respectively, comparison between China and the world. Period RR, period Rate Ratio; cohort RR, cohort Rate Ratio.

Regarding age effect, within the same birth cohort, epilepsy incidence reached its peak in children and the elderly, displaying a similar trend in both China and the global population (Figure 4A). For period effect, global epilepsy incidence steadily increased, whereas in the PRC, it initially experienced rapid growth, even surpassing the global incidence, before slowly decreasing (Figure 4C). Concerning cohort effect, both China and the global population showed a similar increasing trend, with the PRC generally exhibiting higher values than the global cohort effect (Figure 4E). The net drifts in epilepsy incidence for both China and the global population were greater than 0 (0.38 and 0.54%, respectively, *p* < 0.001), with the PRC having a lower net drift than the global net drift. Global local drift increased slowly with age, while the PRC exhibited an initial decrease followed by an increase. In the age group of individuals above 60, the local drift in the PRC was significantly lower than the global local drift (Figure 4G).

### 3.3 Analysis of the mortality trend of epilepsy in the PRC and globally from 1990 to 2019

Mortality rates for epilepsy in all age groups decreased in the PRC in 2019 compared to 1990, particularly among individuals aged 0–4 years. Mortality remained high among the elderly and increased with age (Supplementary Figure S2B). In 2019, the age-standardized mortality rate per 100,000 individuals in the PRC was 0.77 (95%CI: 0.66 to 0.91), significantly lower than the age-standardized incidence rate, and the global rate was similarly low (Supplementary Figure S3B). From 1990 to 2019, both the number and age-standardized rate for both sexes decreased, and the number and ASR of death for males were higher than those for females. Among females, there was a slight increase around 1993–1995, followed by a period of stability before experiencing a rapid decline. In contrast, males exhibited a steady decline (Figure 2B).

Joinpoint regression analysis results revealed that the age-standardized death rate in both China and the global population decreased from 1990 to 2019. The AAPC for China showed a decrease of −2.592% (95%CI: −2.772% to −2.412%), while the global AAPC was −1.004% (95%CI: −1.097% to −0.910%). The rate of decline in the PRC was considerably higher than the global trend. The AAPC for females in the PRC and globally was higher than that for males, indicating that the age-standardized death rate for females decreased at a faster rate than for males (Table 2). Globally, there was a consistent downward trend, while in the PRC, the decrease was more modest during 1990–2000, followed by a relatively stable rate of decline (Figure 3B). Specific APC values for each segment can be found in the Supplementary Table S2.

Concerning age effect, within the same birth cohort, the global mortality rate for epilepsy peaked in children and the elderly. In the PRC, mortality was primarily concentrated among children, with rates considerably higher than the global mortality. The disparity gradually decreased until the age of 30, when it aligned with the global level (Figure 4B). For period effect, epilepsy mortality rates in both China and the global population steadily decreased, with a more pronounced decline in the PRC. Initially, the PRC had higher rates than the global level, but these eventually dropped below the global level (Figure 4D). Regarding cohort effect, global mortality rates first increased and then decreased, while in the PRC, rates gradually decreased from being higher than the global level to slightly lower than the global level (Figure 4F). The net drifts in epilepsy mortality for both China and the global population were less than 0 (−2.33% and − 0.71%, respectively, *p* < 0.001), with the PRC having a lower net drift than the global net drift. Local drifts in the PRC and globally increased slowly, with the PRC consistently exhibiting lower local drifts than the global population (Figure 4H).

### 3.4 Correlation between the burden of epilepsy and the level of socioeconomic development

**Figure 5:**
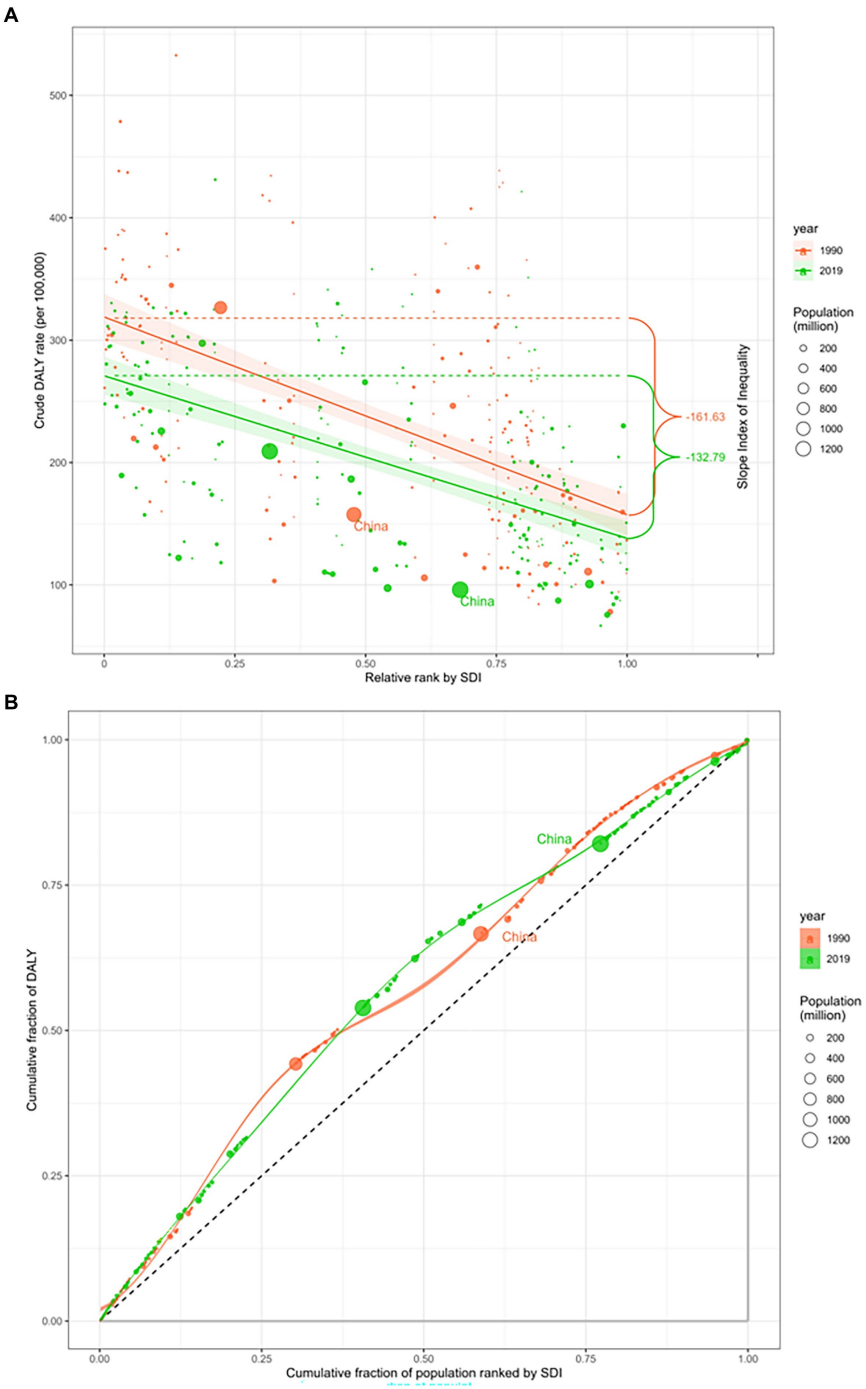
SDI-related health inequality regression **(A)** and concentration **(B)** curves for the burden of epilepsy globally, 1990 and 2019. The size of the circle refers to the size of the population, the red circle marked in the figure represents China in 1990, and the green circle marked in the figure represents China in 2019. SDI, Social demographic index; DALY, Disability-Adjusted life years.

In 1990 and 2019, the global gap in DALYs for epilepsy narrowed, but there were still health inequalities caused by differences due to differences in economic development. SII, which measures health inequality, showed that the disparity in DALYs between countries with the lowest and highest income levels decreased from 161.63 DALYs per 100,000 (95%CI: 129.02 to 194.24) in 1990 to 132.79 DALYs per 100,000 (95%CI: 107.14 to 158.44) in 2019, indicating a reduction in global health inequality (Figure 5A). The RCI, which assesses where health inequalities are concentrated, was −15.37 (95%CI: −16.67 to −14.06) in 1990 and −15.66 (95%CI: −16.86 to −14.46) in 2019. Both values were negative (RCI < 0), indicating that the disease burden of epilepsy in both 1990 and 2019 was concentrated in countries with lower levels of economic development (Figure 5B).

**Figure 6:**
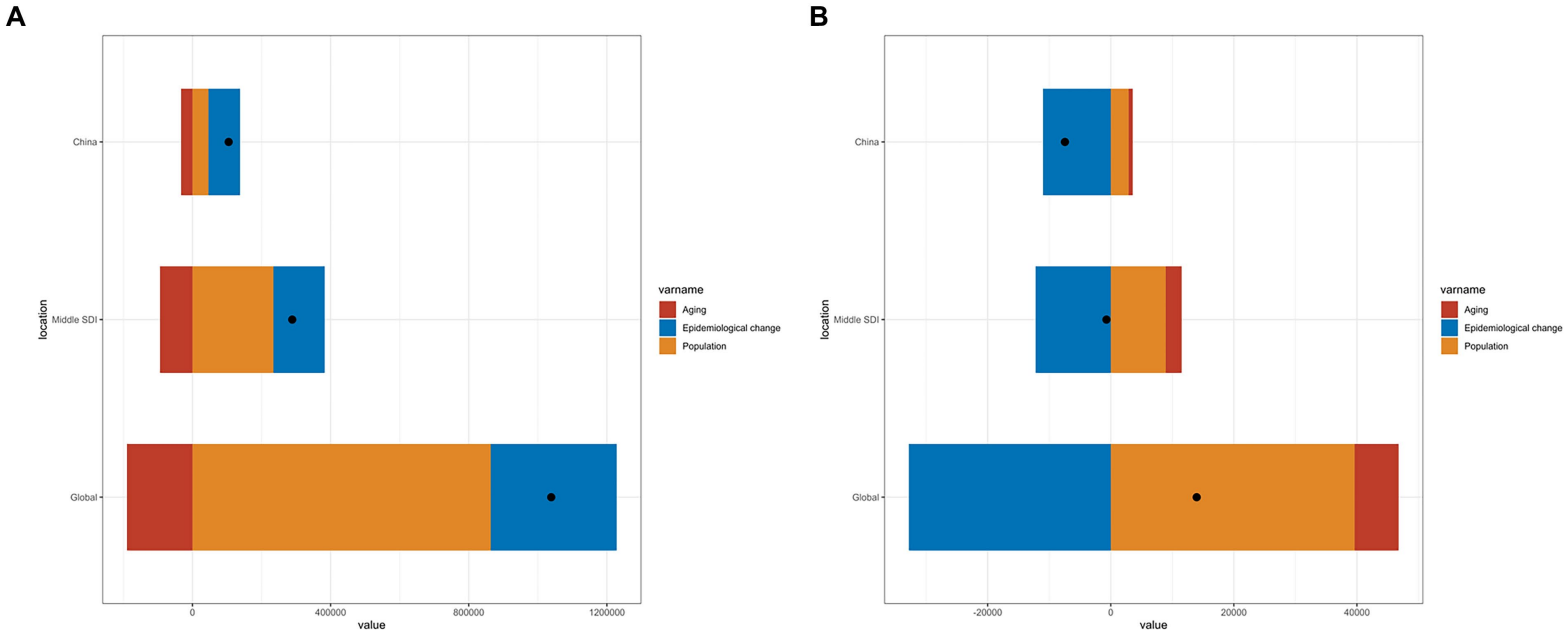
The relative contributions of aging, population, and epidemiological changes to the number of incidence **(A)** and mortality **(B)** from 1990 to 2019, comparison in China, the world, and middle SDI countries. The black dot represents the overall difference of incidence and mortality from 1990 to 2019. SDI, Social demographic index.

Decomposition analysis was employed to quantify the relative contributions of age structure changes, population size changes, and epidemiological changes to the overall burden of epilepsy. The results showed that the increase in the number of epilepsy incidences in the PRC was primarily attributable to underlying age and population-adjusted epilepsy incidence and mortality rates (epidemiological changes), whereas in middle SDI countries and globally, population size changes were the main drivers of increased incidences (Figure 6A). Conversely, the decrease in the number of epilepsy deaths in the PRC was predominantly due to epidemiological changes, while population size changes and epidemiological changes played significant roles in the reduction of deaths in middle SDI countries and globally (Figure 6B; Table 3).

**Table 3:** Change due to population-level determinants (% contribution to the total changes).

Indicator	Location	Overall difference	Aging	Population	Epidemiological change
Incidence	China	104729.05	−33066.57 (−31.57%)	46201.83 (44.12%)	91593.80 (87.46%)
	Middle SDI	288809.70	−94016.41 (−32.55%)	234250.45 (81.11%)	148575.65 (51.44%)
	Global	1038705.39	−189716.23 (−18.26%)	863577.74 (83.14%)	364843.89 (35.12%)
Death	China	−7453.06	675.21 (9.06%)	2891.54 (38.80%)	−11019.81 (−147.86%)
	Middle SDI	−707.35	2596.71 (367.10%)	8918.53 (1260.83%)	−12222.60 (−1727.93%)
	Global	13956.84	7151.62 (51.24%)	39620.20 (283.88%)	−32814.97 (−235.12%)

## 4 Discussion

The primary findings of this retrospective study of the epidemiology of epilepsy from 1990 to 2019, are that the PRC exhibits a higher incidence and lower mortality for epilepsy compared to the global burden, and the burden of epilepsy in the PRC is primarily concentrated among males, children, and the elderly.

In the study, we get to know the burden of epilepsy by using country-specific data sources such as China and comparing it with the global burden. The PRC is a country with a vast territory and abundant resources, and it is also a representative country in the Asian region, so it has an excellent reference value. From 1990 to 2019, with socioeconomic development, the incidence and mortality ratio of epilepsy in the PRC decreased. This decrease can be attributed to an increase in the survival rate (4, 25). When examining the longitudinal age curve, both the incidence and mortality in the PRC and globally displayed similar trends, with notable variations among different age groups, especially concentrated in children and the elderly. In line with our findings, prior studies have indicated that epilepsy’s incidence is highest among young children and the elderly, showcasing a bimodal distribution (15). In children, potential causes of epilepsy may include genetic factors, perinatal ischemia and hypoxia, hippocampal sclerosis, cortical dysplasia, among others (12). Among the elderly, the most widely recognized causes are traumatic injury, alcohol abuse, brain injury, and cerebrovascular diseases (43). Additionally, gender differences were significant, with males bearing a greater burden of epilepsy than females, potentially due to more brain activity, additional medical expenses, and a higher risk of exposure to risk factors (11). Another contributing factor is that consultation rates among females in rural areas were lower due to social stigma and lower economic status (44). It can be seen that we need to implement additional efforts to improve the social status of women to eliminate the risk factors leading to gender differences, especially in rural areas. Considering these age and gender disparities, efforts in epilepsy prevention and treatment should primarily focus on males, children, and the elderly. Furthermore, our research identified that the high DALY for epilepsy occurred globally due to the increased standard life expectancy of patients with improved socioeconomic status (6). In the PRC, the epilepsy burden calculated by DALY has significantly decreased, primarily driven by changes in YLL, indicating a reduction in the loss of life expectancy due to premature death. The China Association Against Epilepsy (CAAE) has established a three-tier system of 201 epilepsy centers, fostering collaboration and information exchange in the PRC (45). This suggests that the reduction in mortality may due to the improved treatment of epilepsy in the PRC (6). The increase in the China’s standard life expectancy has led to an increase in YLD, emphasizing the need to not only reduce premature deaths but also enhance the quality of life for epilepsy patients.

A critical point for incidence and mortality emerged in the 1955–1960 birth cohort in both China and globally. People born after this period exhibited higher incidence and lower mortality, while the opposite pattern was observed among those born before this period. This suggests that younger generations might face a higher risk of developing epilepsy. Given that young people are the backbone of the country and society, focusing on the prevention and treatment of those in later birth cohorts is imperative. In contrast to the continuously increasing global period RR, the period RR of incidence in the PRC gradually decreased after 1997. The period RR of mortality in the PRC and globally also declined similarly, but the decrease in the PRC was faster than the global level. This indicates significant progress in the prevention and treatment of epilepsy in the PRC after 1997, marking an important turning point. In 1997, the WHO, the International League Against Epilepsy (ILAE), and the International Bureau of Epilepsy (IBE) initiated a global campaign against epilepsy called “*Out of the Shadows*” (46). In 2000, a Four-year Demonstration Project was launched in the PRC, training primary health-care physicians in diagnosing and treating epilepsy; thus, reducing disparities in diagnosis and treatment (6). In 2008, the WHO’s Mental Health Gap Action Program aimed at expanding services for mental, neurological, and substance use disorders, including epilepsy (46). In 2011, the Pan American Health Organization (PAHO) Region of the Americas approved the Strategy and Plan of Action on Epilepsy for 2012–2021 (6). In 2015, the World Health Assembly (WHA) in Geneva adopted Resolution WHA68.20 on the Global Burden of Epilepsy. It is the first and only resolution specifically targeting epilepsy in the history of the WHO, which may be the reason why the incidence and age-standardized rate of epilepsy showed a downward trend after 2015 (47). In 2019, the global report on epilepsy titled “*Epilepsy: A Public Health Imperative*” revealed that global investment in epilepsy research is exceedingly insufficient, especially in countries with a low SDI (40, 48). In 2022, the Intersectoral Global Action Plan on Epilepsy and Other Neurological Disorders (IGAP) was approved by the 75^th^ WHA (49). An essential current task is to implement IGAP in the PRC, support the government in formulating “IGAP’s China Program,” collaboratively advance the implementation of IGAP in the PRC, and meet the strategic objectives on schedule.

Inequalities in economic development significantly influence health distribution and access to medical treatment. In comparison to 1990, the global burden of epilepsy in 2019 was concentrated in countries with a low SDI, although health inequalities among countries decreased to some extent. However, only a portion of epilepsy treatment costs are covered by medical insurance, with reimbursements varying greatly depending on diverse national regulations. While countries have developed effective intervention strategies and made some progress, there is a need to allocate health resources rationally, including the allocation of medical resources for epilepsy in different countries and regions of China, such as central, eastern, and western China (50). It is necessary to establish coordinated measures at the country level to improve the health, social, and public implications. The national health system should prioritize the utilization of standardized treatment protocols, enhance the coverage of epilepsy diagnosis and treatment; effectively manage epilepsy-related comorbidities; formulate public health policies that consider national conditions; increase public awareness of epilepsy, and provide psychological education to patients to reduce social stigma (11, 51).

Our study has several limitations. First, the GBD 2019 only provided data at the national and regional levels for disease burden-related indicators, making it impossible to analyze the variability of the data between urban and rural areas in the PRC (4). Second, due to the absence of relevant data, we did not investigate the impact of various risk factors on the epilepsy burden or analyze the effects of comorbidities on the burden of epilepsy. Therefore, our findings should be interpreted cautiously, and further real-world studies are necessary to validate our results. In the future, it is imperative to continually update existing information and incorporate new data sources to achieve more accurate estimates, which could serve as valuable references for health policymakers.

## 5 Conclusion

In summary, the PRC exhibits a higher incidence and lower mortality for epilepsy compared to the global burden, and the disparity in health inequality for epilepsy among countries decreased. The improved survival rate of epilepsy in the PRC between 1990 and 2019 can be largely attributed to epidemiological changes. The burden of epilepsy in the PRC is primarily concentrated among males, children, and the elderly, with younger generations potentially facing a higher risk of developing the condition. In light of these findings, we recommend that the government formulate and execute sensible health policies and preventive measures to eliminate age and gender disparities and safeguard specific populations, especially in low SDI countries.

## Data availability statement

Publicly available datasets were analyzed in this study. This data can be found here: Institute For Health Metrics and Evaluation (IHME), Global Health Data Exchange (GHDx), Global Burden of Disease Study 2019 (GBD 2019), https://ghdx.healthdata.org/gbd-2019.

## Ethics statement

Ethical review and approval was not required for the study on human participants in accordance with the local legislation and institutional requirements. Written informed consent from the patients/participants or patients/participants’ legal guardian/next of kin was not required to participate in this study in accordance with the national legislation and the institutional requirements.

## Author contributions

YS: Data curation, Formal analysis, Writing – original draft. ZW: Data curation, Methodology, Writing – original draft. XY: Data curation, Formal analysis, Writing – review & editing. MS: Data curation, Writing – review & editing. YY: Data curation, Writing – review & editing. CZ: Funding acquisition, Supervision, Writing – review & editing. QY: Supervision, Validation, Writing – review & editing. LW: Methodology, Supervision, Validation, Writing – review & editing.

## Footnotes

^1^ http://ghdx.healthdata.org/

^2^Download site: https://surveillance.cancer.gov/joinpoint/.

^3^ https://vizhub.healthdata.org/gbd-results/

^4^ https://analysistools.cancer.gov/apc/

^5^ https://www.who.int/data/inequality-monitor/assessment_toolkit
