# Supplementary figures and images for: The burden of epilepsy in the People’s Republic of China from 1990 to 2019: epidemiological trends and comparison with the global burden of epilepsy
Source: Front Neurol. 2023 Dec 11;14:1303531. doi: 10.3389/fneur.2023.1303531 (PMC10749336; doi:10.3389/fneur.2023.1303531)

Number

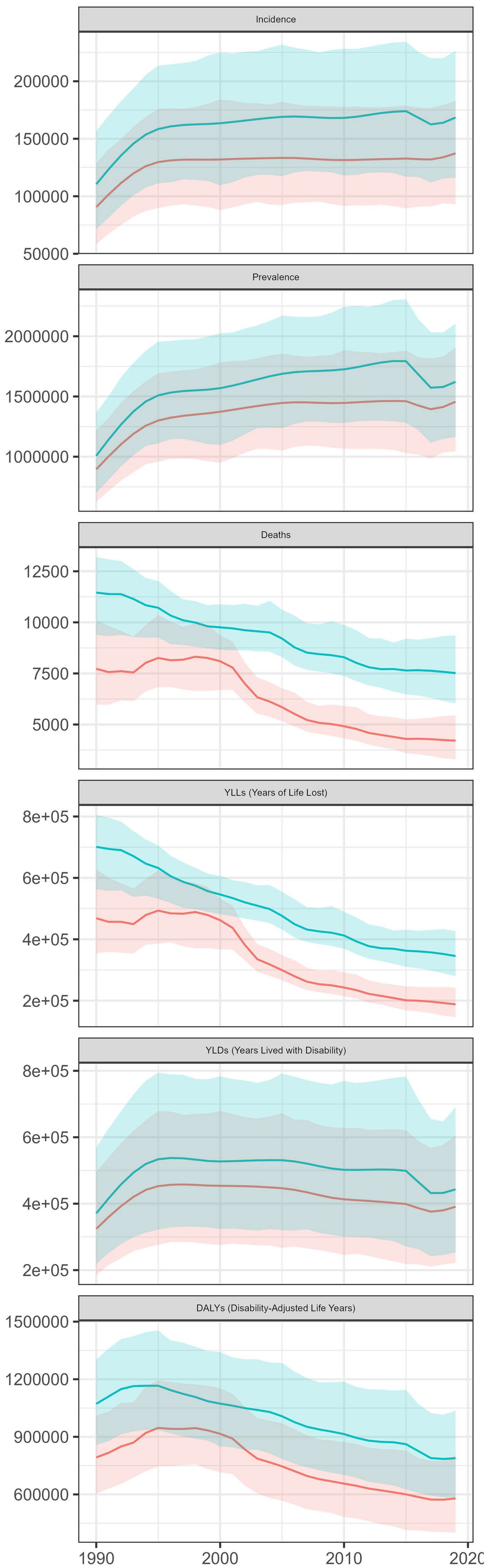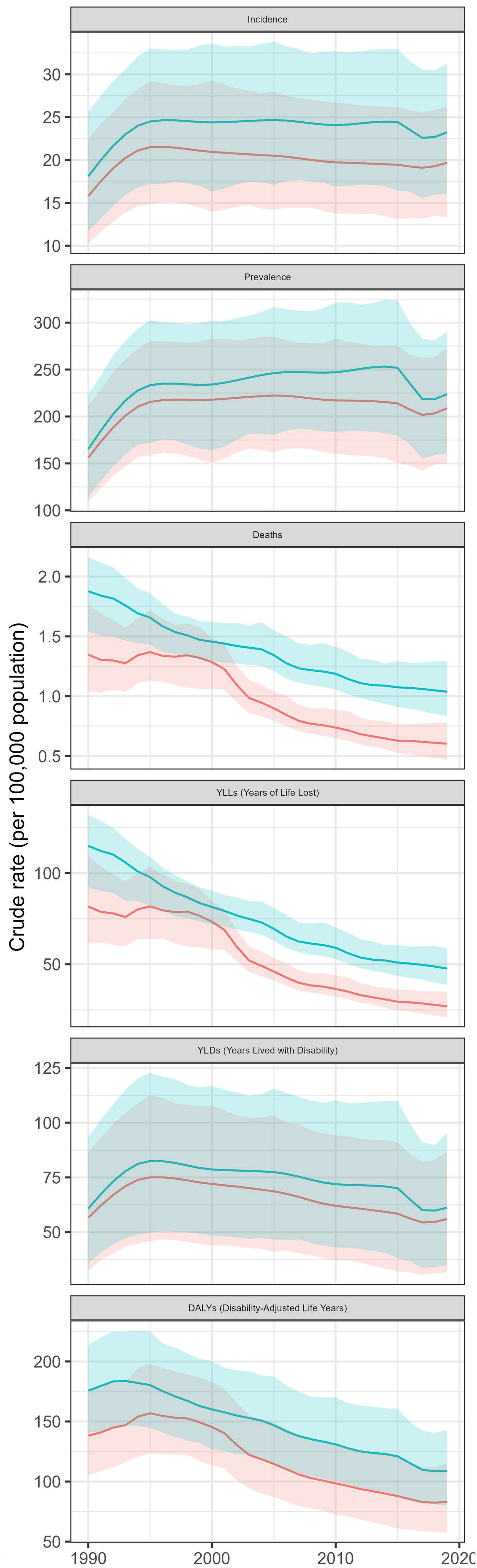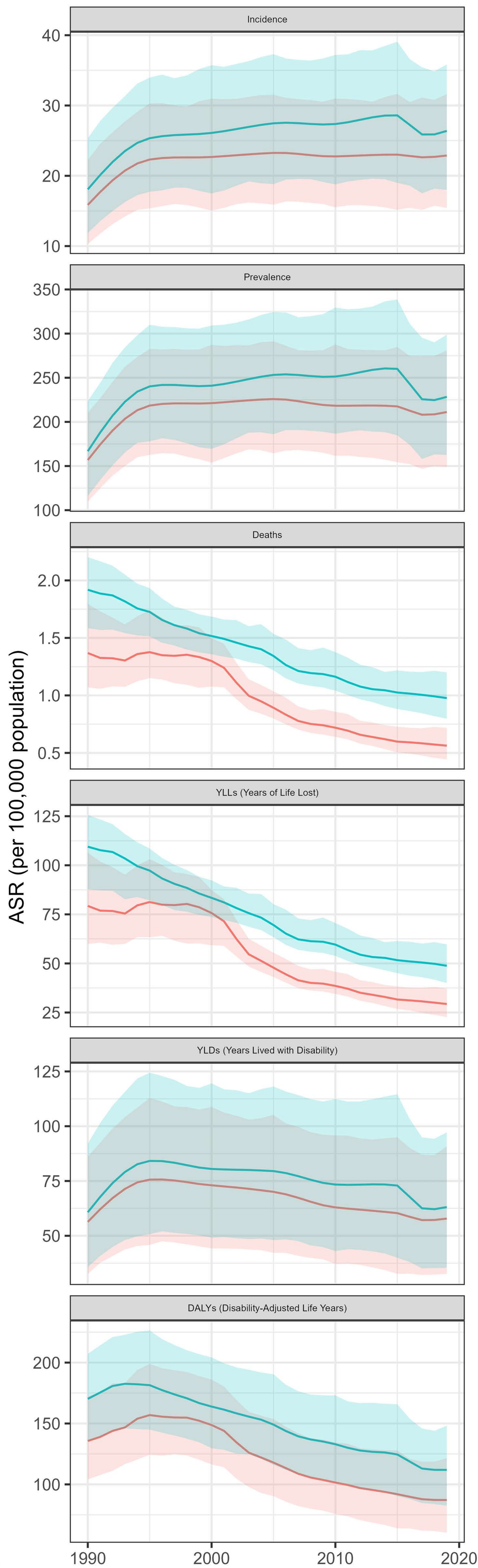

sex

Female

Male

Supplement: Supplementary file 1 [file Data_Sheet_1.PDF]

A

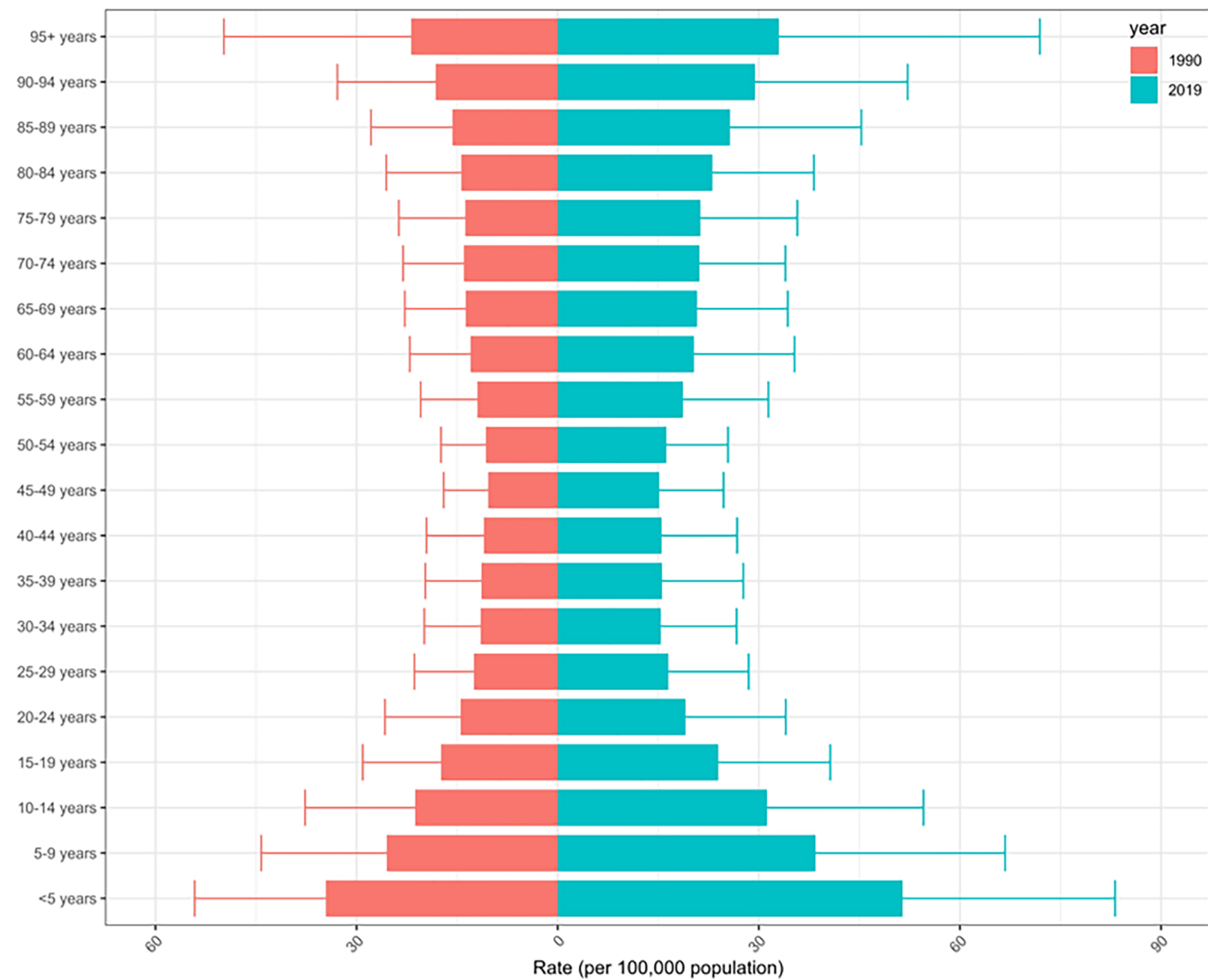

B

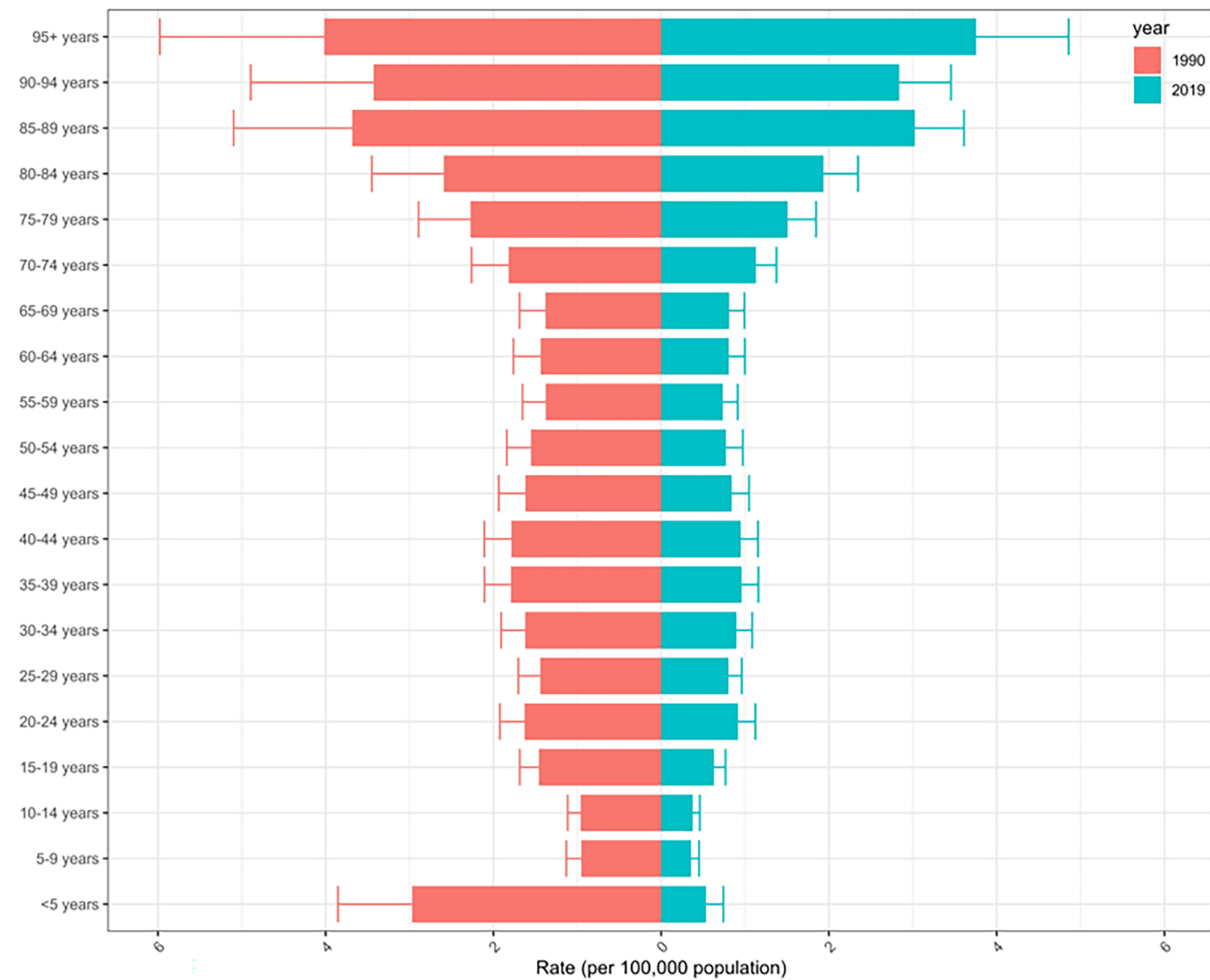

Supplement: Supplementary file 2 [file Data_Sheet_2.PDF]

A

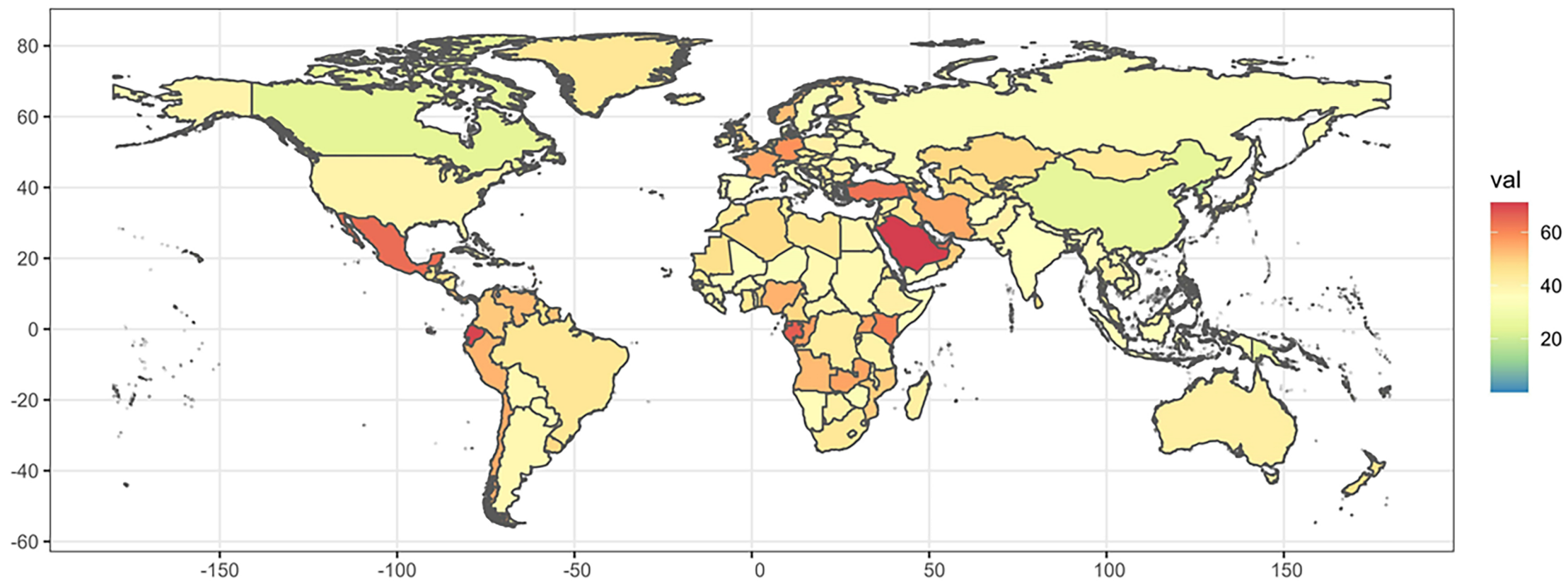

B

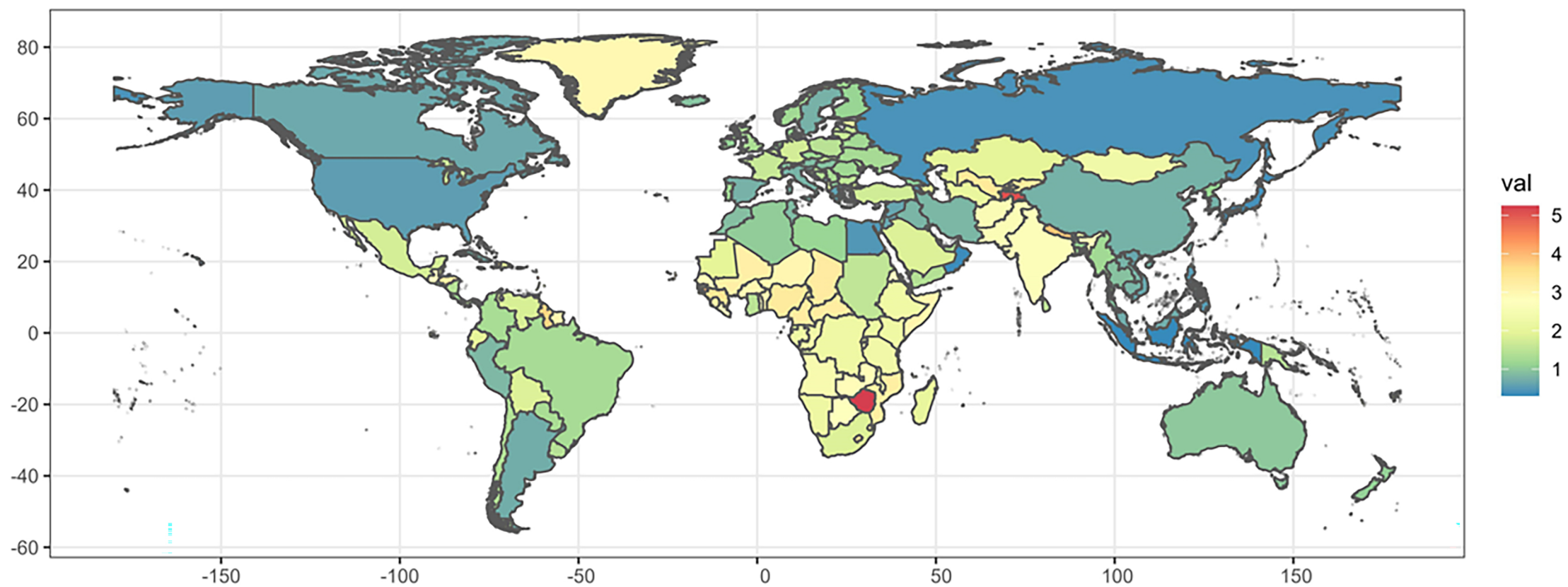

Supplement: Supplementary file 3 [file Data_Sheet_3.PDF]
