# Supplementary material for: The burden of epilepsy in the People’s Republic of China from 1990 to 2019: epidemiological trends and comparison with the global burden of epilepsy
Source: Front Neurol. 2023 Dec 11;14:1303531. doi: 10.3389/fneur.2023.1303531 (PMC10749336; doi:10.3389/fneur.2023.1303531)
Supplement: Supplementary file 6 [file Table_3.DOCX]

**Figure Legends**

**Additional file 1.** The trends in the number, crude rate, and ASR of the incidence, prevalence, death, DALY, YLD, and YLL for epilepsy in Chinese males and females from 1990 to 2019. The red and green regions represent 95% CI. ASR, Age-standardized rate; DALY, Disability-Adjusted life years; YLD, Years lived with disability; YLL, Years of life lost.

**Additional file 2.** The incidence (A) and mortality (B) rate of epilepsy for different age groups in China, 1990 and 2019.

**Additional file 3.** The world map of the age-standardized incidence (A) and mortality (B) rate of epilepsy for 204 countries in 2019. The color bar on the right refers to the value from low to high.
